# Supplementary material for: Deployment of endocytic machinery to periactive zones of nerve terminals is independent of active zone assembly and evoked release
Source: bioRxiv. 2026 Feb 26:2025.04.23.650151. Preprint. [Version 2] doi: 10.1101/2025.04.23.650151 (PMC13160045; doi:10.1101/2025.04.23.650151)

bioRxiv preprint doi: https://doi.org/10.1101/2025.04.23.650151; this version posted February 26, 2026. The copyright holder for this preprint (which was not certified by peer review) is the author/funder, who has granted bioRxiv a license to display the preprint in perpetuity. It is made available under aCC-BY 4.0 International license.

Figure supplements

Figure 1 - figure supplement 1. Confocal microscopic analyses of synapses after chronic silencing or acute depolarization of mouse hippocampal neurons

(**A, B**) Example confocal images (A) and quantification of the average intensities (B) of Amphiphysin, PIPK1γ, AP-180, Dynamin-1, Bassoon and Munc13-1 at synapses identified as Synaptophysin puncta. Intensities are normalized to the average signals in the untreated conditions per culture; n in B (images/cultures): Amphiphysin, 14/3; PIPK1γ, untreated 14/3, blockers 14/3, KCl 13/3; AP-180, 15/3; Dynamin-1, 14/3; Synaptophysin, untreated 57/3, blockers 57/3, KCl 56/3; Bassoon, untreated 43/3, blockers 43/3, KCl 42/3; Munc13-1, 14/3. The increase in Munc13-1 upon chronic silencing, which we previously reported (Held et al., 2020), and of Synaptophysin, may reflect a homeostatic adaptation. The decrease in Amphiphysin upon KCl stimulation may reflect a redistribution of this protein during prolonged stimulation. Data are mean ± SEM; *$p < 0.05$, **$p < 0.05$, ***$p < 0.001$ compared to the untreated condition determined by one-way ANOVA followed by a Tukey-Kramer post hoc tests for Bassoon or Kruskal-Wallis followed by Holm post hoc tests for Amphiphysin, PIPK1γ, AP-180, Dynamin-1, Synaptophysin and Munc13-1.

Figure 1 - figure supplement 2. Workflows for STED analyses in mouse hippocampal neurons and for confocal analyses at *Drosophila* neuromuscular junctions

(**A**) Workflow for the analyses of side-view synapses of mouse hippocampal neurons, showing an example synapse immunostained for the active zone marker Bassoon (imaged in STED), PIPK1γ (imaged in STED) and Synaptophysin (imaged in confocal). Synapse selection and placement of an area of interest (white rectangle with line profile direction indicated by arrow) perpendicular to the marker (Bassoon, in this example) is done by an experimenter blind for the protein of interest (PIPK1γ, in this example). Next, the protein of interest channel is activated,

bioRxiv preprint doi: https://doi.org/10.1101/2025.04.23.650151; this version posted February 26, 2026. The copyright holder for this preprint (which was not certified by peer review) is the author/funder, who has granted bioRxiv a license to display the preprint in perpetuity. It is made available under aCC-BY 4.0 International license.

and the profile is generated. Finally, the average line profiles, peak intensities and distance of proteins of interest to the marker are plotted.

(**B**) Workflow for the analyses of en-face synapses of mouse hippocampal neurons, showing an example synapse immunostained for Bassoon (imaged in STED), PIPK1γ (imaged in STED) and Synaptophysin (imaged in confocal). First, synapse selection is done by an experimenter blind for the protein of interest. Next, the channel of the protein of interest is activated, and objects containing endocytic proteins and the marker are identified in the respective channels using an algorithm. Finally, the number of objects per synapse, their lateral distance to the active zone, and their integrated intensity are plotted.

(**C**) Workflow for analyses of *Drosophila* neuromuscular junctions. Terminals are analyzed both in 3D and in 2D half-maximum intensity projections. First, the average intensities of the active zone marker (Brp in this example) and the endocytic protein (Nervous Wreck in this example) are quantified in the full 3D volume of the terminal. Next, the periactive zone levels and degree of polarization are analyzed in 2D half-maximum intensity projections. The polarization of each protein is quantified as the ratio between its average intensity at the mesh over its average intensity in the core. To conduct this analysis, segmentation into mesh and core is performed based on the difference in signal between proteins enriched in the periactive zone mesh (e.g. Nwk and Dynamin) vs. proteins enriched in the core region (e.g. Brp and Pak) as described in the methods. White lines delineate the center of the mesh regions. The mesh is ~200 nm wide, and the core is the remaining enclosed region within the innermost bounds. The resulting ROIs are used to measure average intensities within the mesh and the core and its ratio.

Figure 1 - figure supplement 3. Assessment of AP-180 with alternate antibody after chronic silencing or acute depolarization of mouse hippocampal neurons

(**A, B**) Example side-view synapses (A) and average line profiles of AP-180 (antibody A246) and Munc13-1 (b). Neurons were stained for AP-180 (imaged in STED), Munc13-1 (imaged in

bioRxiv preprint doi: https://doi.org/10.1101/2025.04.23.650151; this version posted February 26, 2026. The copyright holder for this preprint (which was not certified by peer review) is the author/funder, who has granted bioRxiv a license to display the preprint in perpetuity. It is made available under aCC-BY 4.0 International license.

STED), and the synaptic vesicle marker Synaptophysin (imaged in confocal). An area of interest was positioned perpendicular to the center of the Munc13-1 object, and synapses were aligned via the peak fluorescence of Munc13-1 in the average profiles. Line profiles were normalized to the average signal in the untreated condition. Dashed lines mark average levels in the untreated condition and grey shaded areas represent the active zone area; n in B (synapses/cultures): untreated, 58/3; blockers, 61/3; KCl, 50/3.

(C, D) Quantification of the peak-to-peak distance of the active zone marker and the protein of interest (C), and of the peak levels in the periactive zone area (D). The periactive zone area is defined as an area within 68 nm on each side of the peak of the active zone marker (grey shaded areas in B); n as in B.

Data are mean ± SEM; *p < 0.05, ***p < 0.001 compared to the untreated condition determined by Kruskal-Wallis followed by Holm post-hoc tests.

Figure 1 - figure supplement 4. Additional analyses of en-face synapses after chronic silencing or acute depolarization of mouse hippocampal neurons

Quantification of the average integrated intensities (calculated as the object area multiplied by its average fluorescence intensity) of the Amphiphysin, PIPK1γ, AP-180 and Dynamin-1 objects detected in en-face synapses from Fig. 1L-Q. Intensities are normalized to the average signals in the untreated conditions per culture; n as in Fig. 1P.

Data are mean ± SEM; **p < 0.01, ***p < 0.001 compared to the untreated condition determined by one-way ANOVA followed by a Tukey-Kramer post hoc test for AP-180 or Kruskal-Wallis followed by Holm post hoc tests for Amphiphysin, PIPK1γ and Dynamin-1.

Figure 2 - figure supplement 1. Validation of EndoA, Dap160, and Dynamin antibodies in *Drosophila* NMJs. Example confocal images and quantification of the signal of EndoA (A+B), Dap160 (C+D) or Dynamin (E+F) in NMJs expressing either driver alone (control) or an RNAi

bioRxiv preprint doi: https://doi.org/10.1101/2025.04.23.650151; this version posted February 26, 2026. The copyright holder for this preprint (which was not certified by peer review) is the author/funder, who has granted bioRxiv a license to display the preprint in perpetuity. It is made available under aCC-BY 4.0 International license.

against the indicated gene. Data are expressed as the percentage of the control; n in B (NMJs/animal): control 15/3, EndoA-RNAi 14/3; D: control 3/2, Dap160-RNAi 3/2; F: control 19/6, Dyn-RNAi 38/6. Data are mean ± SEM.

Figure 3 - figure supplement 1. Assessment of EndoA and Dap160 after chronic silencing of *Drosophila* NMJs using STED microscopy

Example boutons with or without TeNT-expression (A), and quantification of the average fluorescence intensity of EndoA and Dap160 per bouton (B), average intensity at the periactive zone mesh (C) and the polarization within periactive zone units (D). Data in B and C are normalized to the average of the control condition; n in B (NMJ/animal): control 20/6, TeNT 16/6; C-D: control 20/6, TeNT 15/6.

Data are mean ± SEM; *p < 0.05 determined by two-sided Student's t-tests. Images acquired by STED microscopy.

Figure 4 - figure supplement 1. Confocal microscopic analyses of synapses after Ca_V2 ablation in mouse hippocampal neurons

(**A, B**) Example confocal images (A) and quantification of the average intensities (B) of Amphiphysin, PIPK1γ, AP-180 and Dynamin-1 at synapses identified as Synaptophysin puncta. Intensities are normalized to the average signals in the control^{Cav2} condition per culture; n in B (images/cultures): Amphiphysin 20/3, PIPK1γ 20/3, AP-180 17/3, Dynamin-1 18/3, Bassoon 57/3, Munc13-1 18/3, Synaptophysin 75/3.

Data are mean ± SEM; *p < 0.05 determined by a two-sided Student's t-tests for Amphiphysin, PIPK1γ, Bassoon and Munc13-1, or two-sided Mann-Whitney U tests for AP-180, Dynamin-1 and Synaptophysin.

Figure 4 - figure supplement 2. Assessment of AP-180 with an alternate antibody after

bioRxiv preprint doi: https://doi.org/10.1101/2025.04.23.650151; this version posted February 26, 2026. The copyright holder for this preprint (which was not certified by peer review) is the author/funder, who has granted bioRxiv a license to display the preprint in perpetuity. It is made available under aCC-BY 4.0 International license.

Ca_V2 ablation in mouse hippocampal neurons

(**A, B**) Example side-view synapses (A) and average line profiles (B) of AP-180 (antibody A246) and Munc13-1. Neurons were stained for AP-180 (imaged in STED), Munc13-1 (imaged in STED), and the synaptic vesicle marker Synaptophysin (imaged in confocal). An area of interest was positioned perpendicular to the center of the Munc13-1 object, and synapses were aligned via the peak fluorescence of Munc13-1 in the average profiles. Line profiles were normalized to the average signal in $control^{Cav2}$ condition. Dashed lines mark average levels in the $control^{Cav2}$ and grey shaded areas represent the active zone area; n in B (synapses/cultures): $control^{Cav2}$ 50/3, $cTKO^{Cav2}$ 48/3.

(C, D) Quantification of the peak-to-peak distance of Munc13-1 and AP-180 (C) and of their peak levels in the periactive zone area (D). The periactive zone area is defined as an area within 68 nm on each side of the peak of the active zone marker (grey shaded areas in B); n as in B.

Data are mean ± SEM; $^*p < 0.05$, shown compared to the $control^{Cav2}$ determined by a two-sided Student's t-test (D for AP-180) or two-sided Mann-Whitney U tests (C,D for Munc13-1).

Figure 4 - figure supplement 3. Additional analyses of en-face synapses after Ca_V2 ablation in mouse hippocampal neurons

Quantification of the average integrated intensities (calculated as the object area multiplied by its average fluorescence intensity) of the Amphiphysin, PIPK1γ, AP-180 and Dynamin-1 objects detected in the en-face synapses quantified in Fig. 3L-Q. Intensities are normalized to the average signals in $control^{Cav2}$ per culture; n as in Fig. 3P.

Data are mean ± SEM; $^*p < 0.05$ determined by Mann-Whitney U tests.

Figure 5 - figure supplement 1. Additional analyses of endocytic proteins after active zone disruption in mouse hippocampal neurons

bioRxiv preprint doi: https://doi.org/10.1101/2025.04.23.650151; this version posted February 26, 2026. The copyright holder for this preprint (which was not certified by peer review) is the author/funder, who has granted bioRxiv a license to display the preprint in perpetuity. It is made available under aCC-BY 4.0 International license.

(**A, B**) Example confocal images (A) and quantification of the average intensities (B) of Amphiphysin, PIPK1γ, AP-180 and Dynamin-1 at synapses identified as Synaptophysin or Synapsin puncta. Intensities are normalized to the average signals in control^{R+E} per culture; n in B (images/cultures): Amphiphysin, control^{R+E} 15/3, cQKO^{R+E} 16/3; PIPK1γ, 16/3; AP-180, 16/3; Dynamin-1, control^{R+E} 18/3, cQKO^{R+E} 19/3; PSD-95, control^{R+E} 65/3, cQKO^{R+E} 67/3; Synaptophysin, control^{R+E} 47/3, cQKO^{R+E} 48/3; Synapsin, control^{R+E} 18/3, cQKO^{R+E} 19/3. Data are mean ± SEM; *$p < 0.05$, ***$p < 0.001$ determined by two-sided Student's t tests for Dynamin-1 and Synapsin or by two-sided Mann-Whitney U tests for Amphiphysin, PIPK1γ, AP-180, PSD-95 and Synaptophysin.

Figure 5 - figure supplement 2. Assessment of AP-180 with an alternate antibody after active zone disruption in mouse hippocampal neurons

(**A, B**) Example side-view synapses (A) and average line profiles (B) of AP-180 (antibody A246) and PSD-95. Neurons were stained for AP-180 (imaged in STED), PSD-95 (imaged in STED), and the synaptic vesicle marker Synaptophysin (imaged in confocal). A line profile was positioned perpendicular to the center of the PSD-95 object, and synapses were aligned via the peak fluorescence of PSD-95 in the average profiles. Line profiles were normalized to the average signal in control^{R+E}. Dashed lines mark average levels in the control^{R+E} condition and grey shaded areas represent the active zone area; n in b (synapses/cultures): control^{R+E} 52/3, cQKO^{R+E} 46/3.

(C, D) Quantification of the peak-to-peak distance of PSD-95 and AP-180 (C) and of their peak levels in the periactive zone area (D). The periactive zone area is defined as the area -136 nm from the PSD-95 peak towards the presynaptic bouton (grey shaded areas in B); n as in B. Data are mean ± SEM; *$p < 0.05$, shown compared to the control^{Cav2} condition determined by a two-sided Student's t-test (D for AP-180) or two-sided Mann-Whitney U tests (C, D for Munc13-1).

bioRxiv preprint doi: https://doi.org/10.1101/2025.04.23.650151; this version posted February 26, 2026. The copyright holder for this preprint (which was not certified by peer review) is the author/funder, who has granted bioRxiv a license to display the preprint in perpetuity. It is made available under aCC-BY 4.0 International license.

Figure 5 - figure supplement 3. Additional analyses of en-face synapses after active zone disruption in mouse hippocampal neurons

Quantification of the average integrated intensities (calculated as the object area multiplied by its average fluorescence intensity) of the Amphiphysin, PIPK1γ, AP-180 and Dynamin-1 objects detected in the en-face synapses quantified in Fig. 5M-R. Intensities are normalized to the average signal in control^{R+E} per culture, n as in Fig. 5Q.

Data are mean ± SEM; **p < 0.01 ***p < 0.001 determined by two-sided Mann-Whitney U tests.

Figure 7 - figure supplement 1. Confocal microscopic analyses of synapses after Liprin-α ablation in mouse hippocampal neurons

(**A, B**) Example confocal images (A) and quantification of the average intensities (B) of Amphiphysin, PIPK1γ, AP-180 and Dynamin-1 at synapses identified as Synaptophysin or Synapsin puncta. Intensities are normalized to the average signal in control^{L1-4} per culture; n in B (images/cultures): Amphiphysin 26/3, PIPK1γ 16/3, AP-180 20/3, Dynamin-1 18/3, PSD-95 80/3, Synaptophysin 62/3, Synapsin 18/3.

Data are mean ± SEM; *p < 0.05 determined by two-sided Student's t-tests for Synapsin and Dynamin-1 or two-sided Mann-Whitney U tests for Amphiphysin, PIPK1γ, AP-180, PSD-95 and Synaptophysin.

Figure 7 - figure supplement 2. Additional analyses of en-face synapses after Liprin-α ablation in mouse hippocampal neurons

Quantification of the average integrated intensities (calculated as the object area multiplied by its average fluorescence intensity) of the Amphiphysin, PIPK1γ, AP-180 and Dynamin-1 objects detected in the en-face synapses quantified in Fig. 7M-R. Intensities are normalized to the average signals in control^{L1-4} per culture; n as in Fig. 7Q.

bioRxiv preprint doi: https://doi.org/10.1101/2025.04.23.650151; this version posted February 26, 2026. The copyright holder for this preprint (which was not certified by peer review) is the author/funder, who has granted bioRxiv a license to display the preprint in perpetuity. It is made available under aCC-BY 4.0 International license.

Data are mean ± SEM; statistical significance was assessed by two-sided Mann-Whitney U tests.

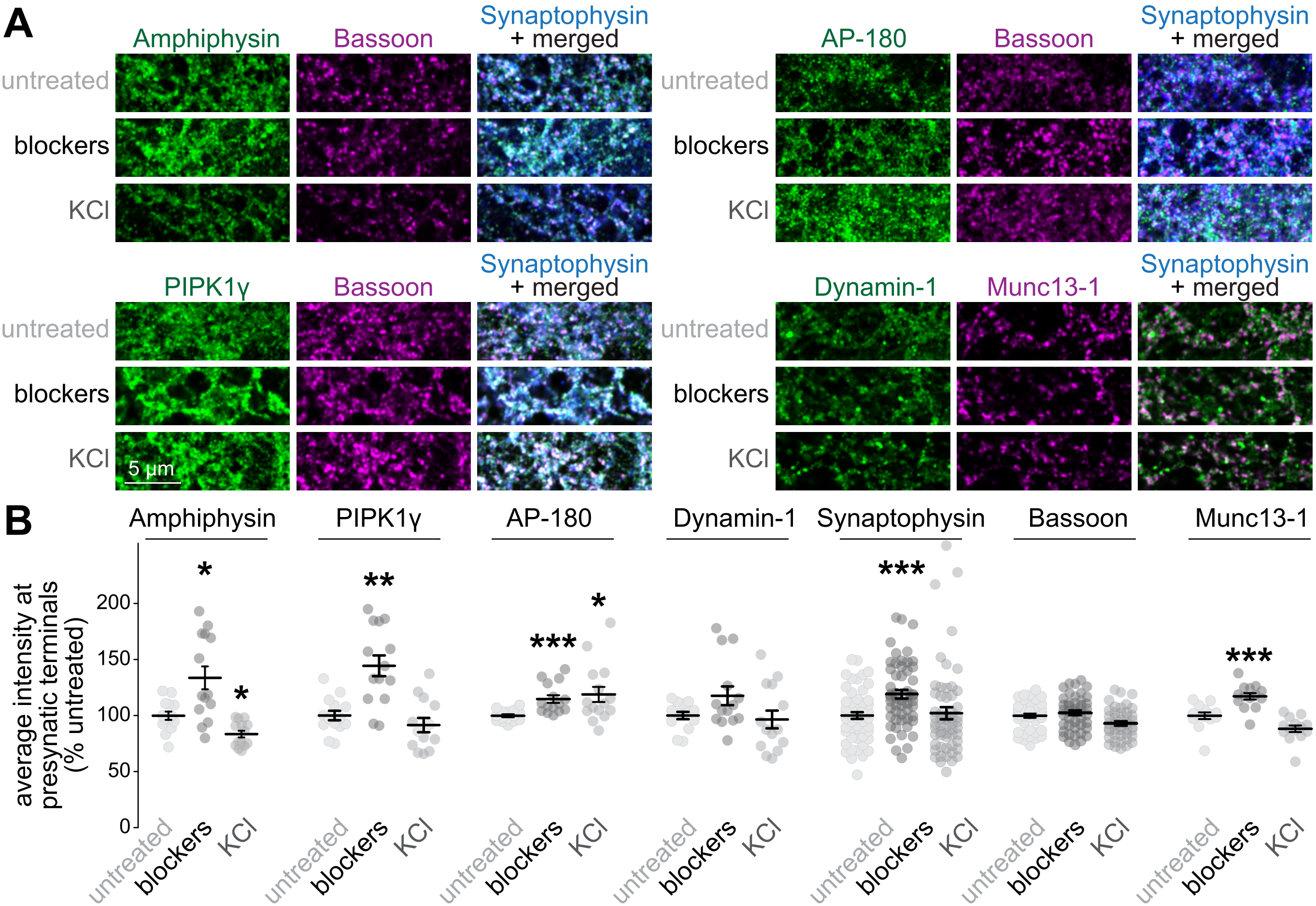

A
Amphiphysin
Bassoon
Synaptophysin
+ merged
AP-180
PIPK1γ
Dynamin-1
Munc13-1
untreated
blockers
KCl
5 μm
B
Synaptophysin
average intensity at presynaptic terminals (% untreated)
0
50
100
150
200
*
**
***

bioRxiv preprint doi: https://doi.org/10.1101/2025.04.23.650151; this version posted February 26, 2026. The copyright holder for this preprint (which was not certified by peer review) is the author/funder, who has granted bioRxiv a license to display the preprint in perpetuity. It is made available under aCC-BY 4.0 International license.

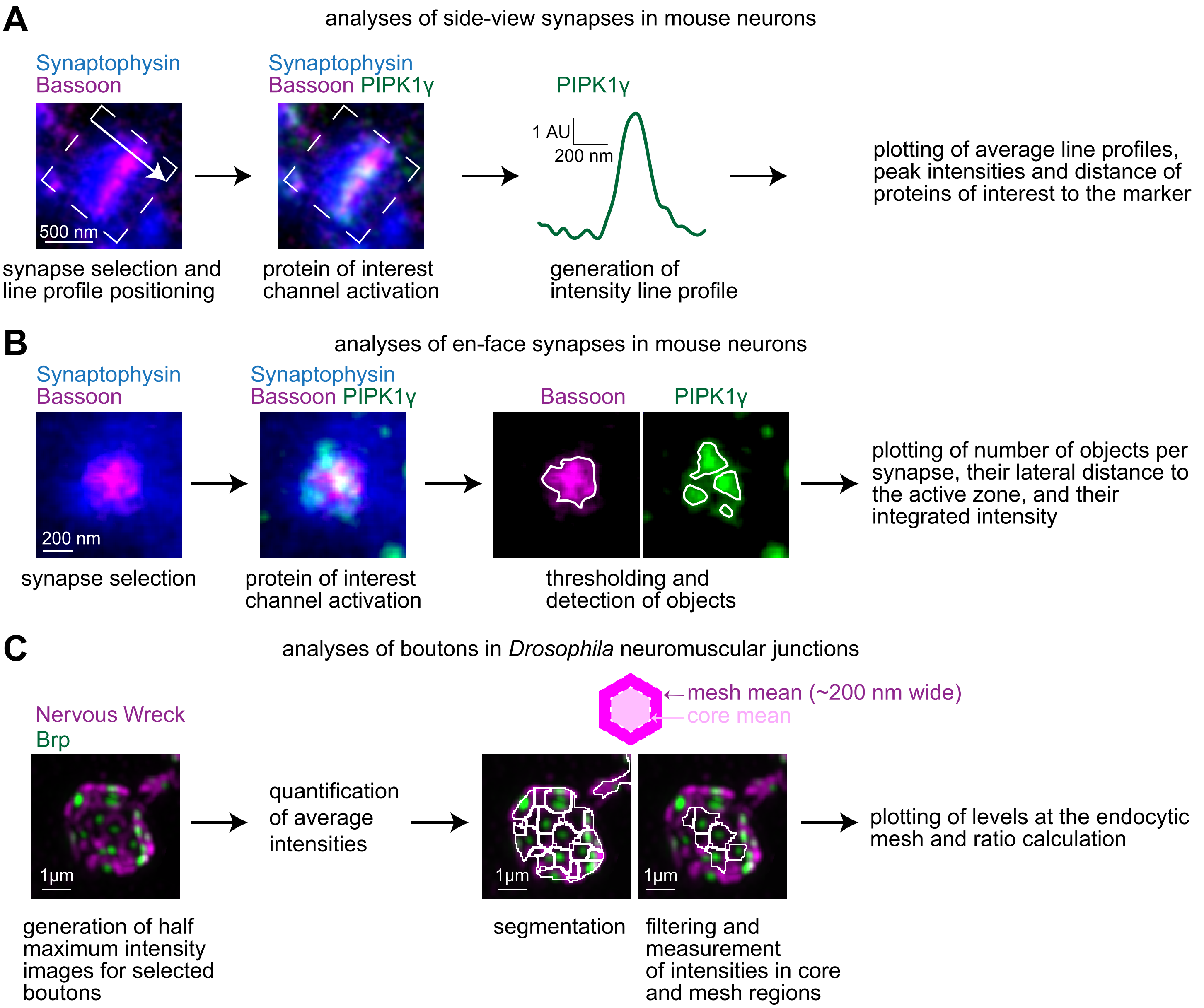

bioRxiv preprint doi: https://doi.org/10.1101/2025.04.23.650151; this version posted February 26, 2026. The copyright holder for this preprint (which was not certified by peer review) is the author/funder, who has granted bioRxiv a license to display the preprint in perpetuity. It is made available under aCC-BY 4.0 International license.

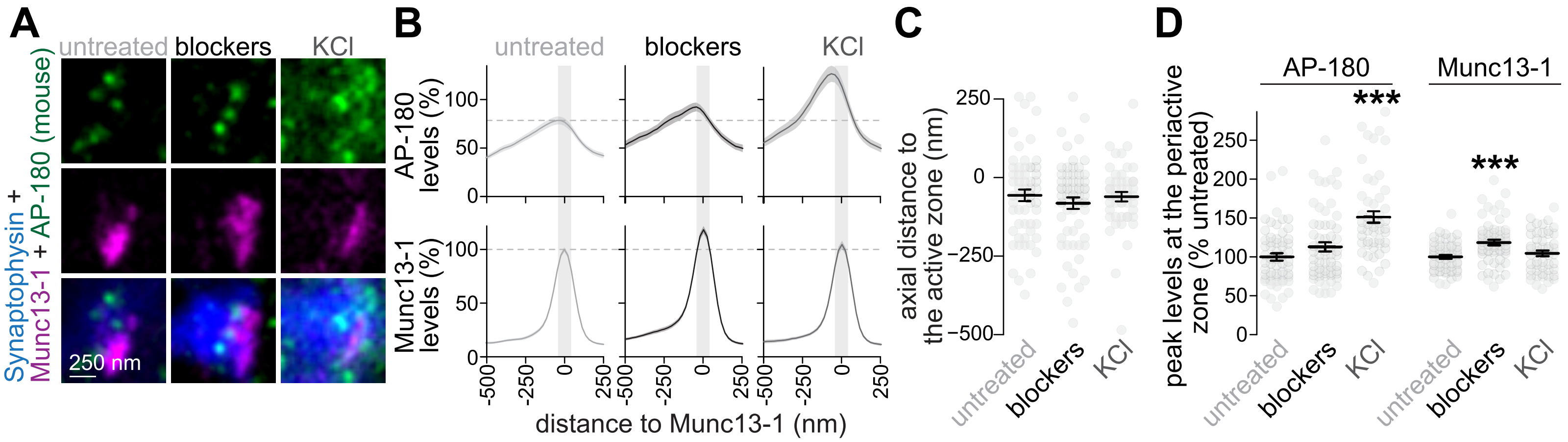

bioRxiv preprint doi: https://doi.org/10.1101/2025.04.23.650151; this version posted February 26, 2026. The copyright holder for this preprint (which was not certified by peer review) is the author/funder, who has granted bioRxiv a license to display the preprint in perpetuity. It is made available under aCC-BY 4.0 International license.

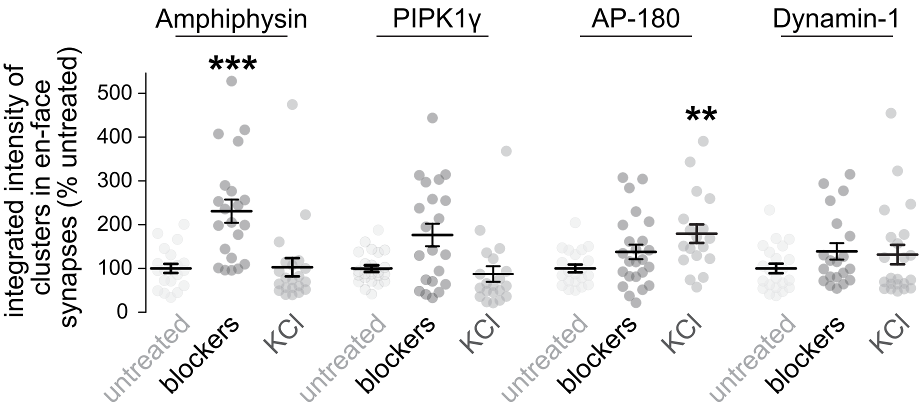

bioRxiv preprint doi: https://doi.org/10.1101/2025.04.23.650151; this version posted February 26, 2026. The copyright holder for this preprint (which was not certified by peer review) is the author/funder, who has granted bioRxiv a license to display the preprint in perpetuity. It is made available under aCC-BY 4.0 International license.

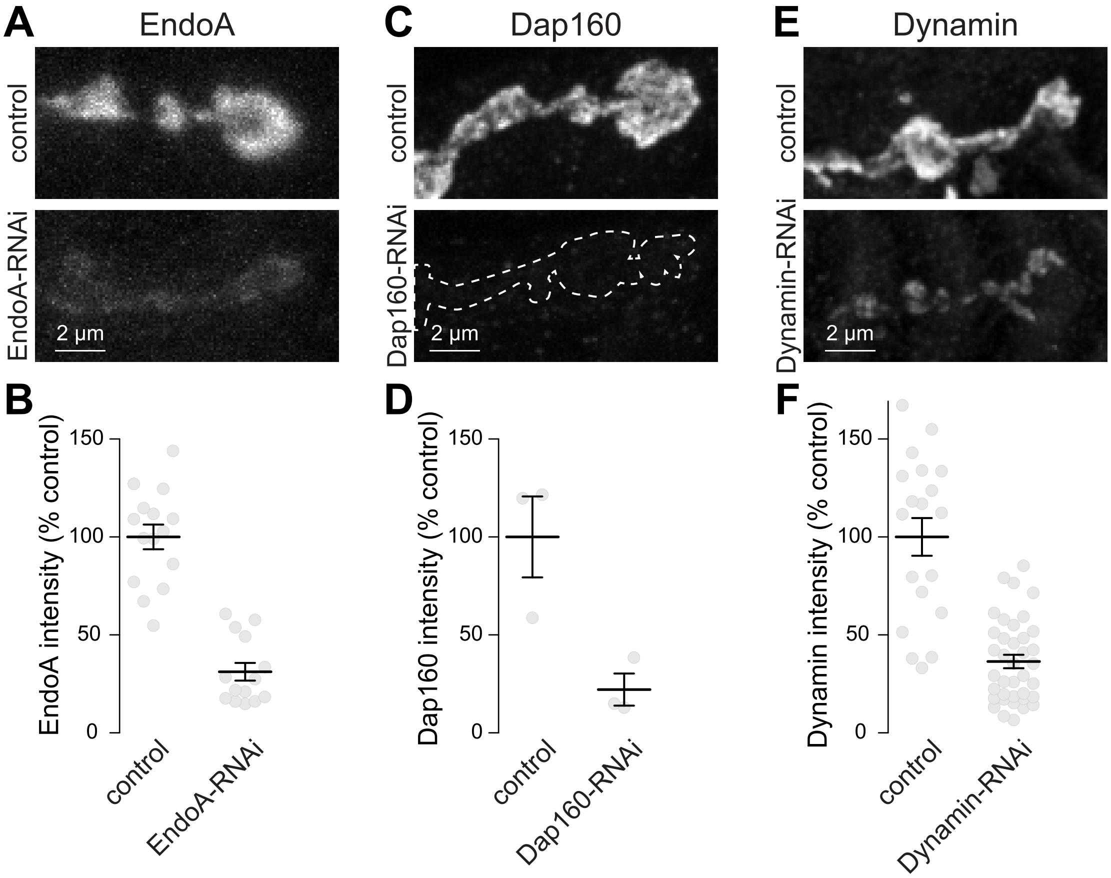

bioRxiv preprint doi: https://doi.org/10.1101/2025.04.23.650151; this version posted February 26, 2026. The copyright holder for this preprint (which was not certified by peer review) is the author/funder, who has granted bioRxiv a license to display the preprint in perpetuity. It is made available under aCC-BY 4.0 International license.

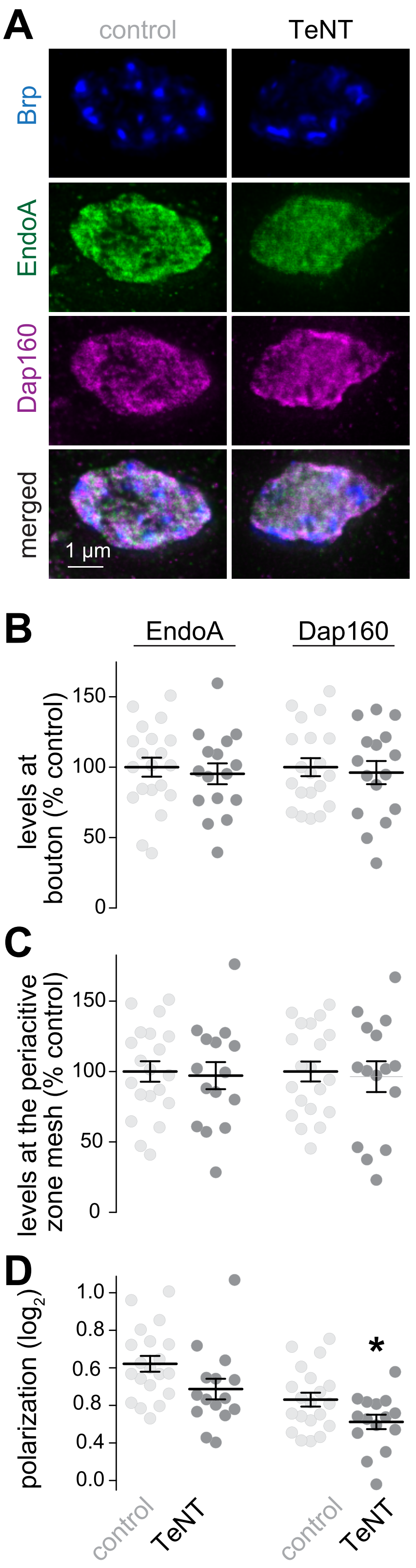

bioRxiv preprint doi: https://doi.org/10.1101/2025.04.23.650151; this version posted February 26, 2026. The copyright holder for this preprint (which was not certified by peer review) is the author/funder, who has granted bioRxiv a license to display the preprint in perpetuity. It is made available under aCC-BY 4.0 International license.

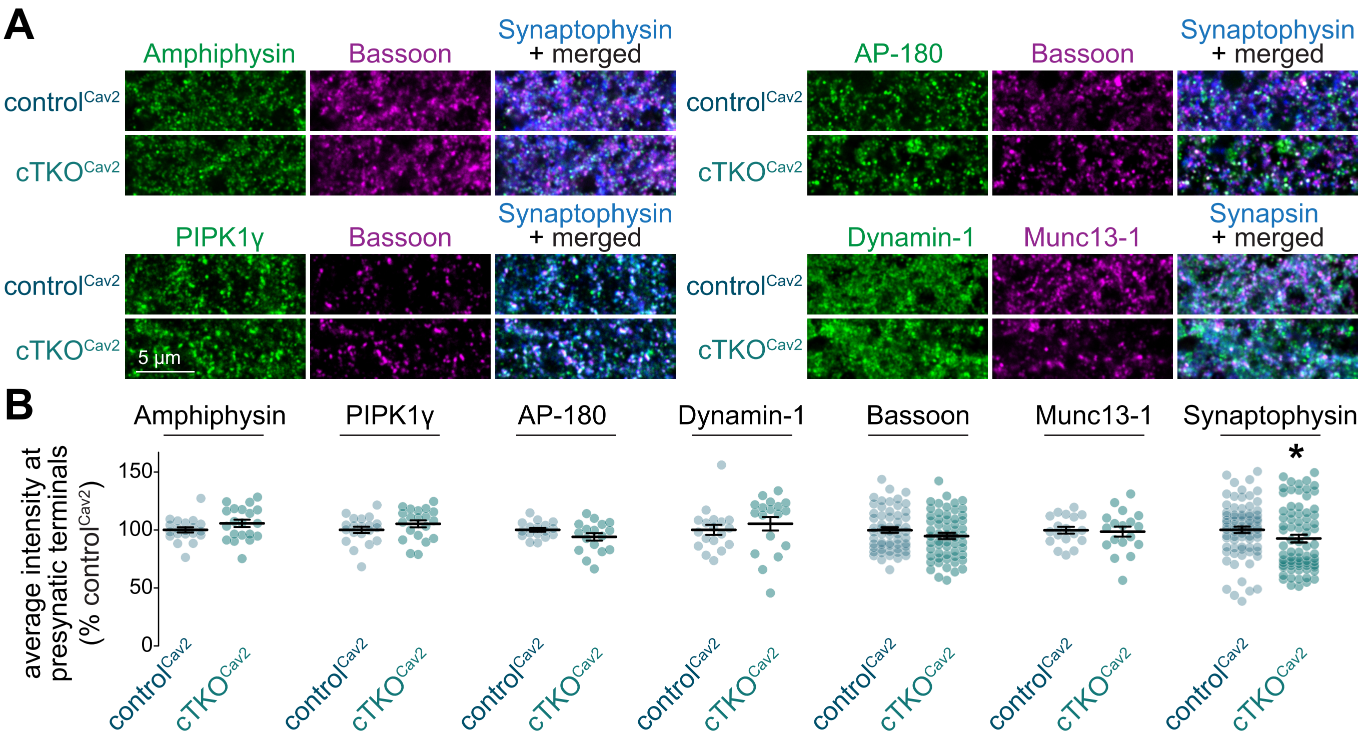

bioRxiv preprint doi: https://doi.org/10.1101/2025.04.23.650151; this version posted February 26, 2026. The copyright holder for this preprint (which was not certified by peer review) is the author/funder, who has granted bioRxiv a license to display the preprint in perpetuity. It is made available under aCC-BY 4.0 International license.

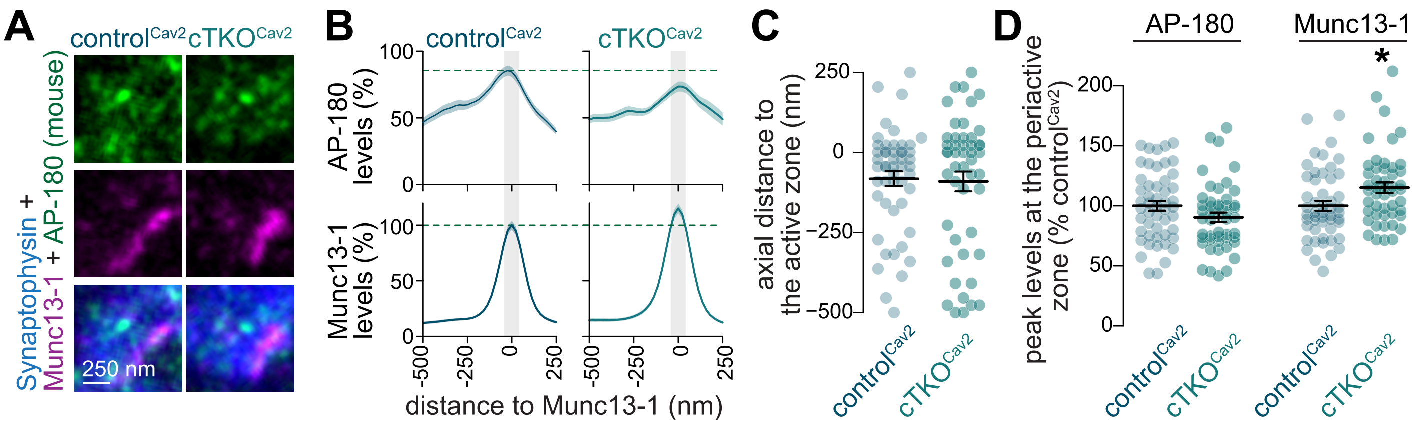

bioRxiv preprint doi: https://doi.org/10.1101/2025.04.23.650151; this version posted February 26, 2026. The copyright holder for this preprint (which was not certified by peer review) is the author/funder, who has granted bioRxiv a license to display the preprint in perpetuity. It is made available under aCC-BY 4.0 International license.

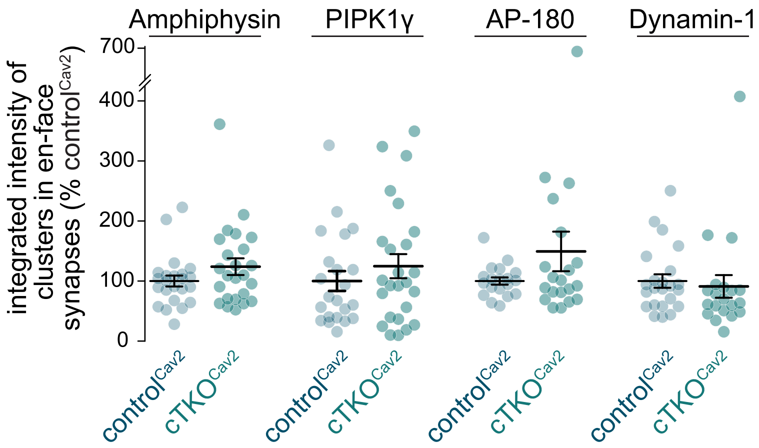

bioRxiv preprint doi: https://doi.org/10.1101/2025.04.23.650151; this version posted February 26, 2026. The copyright holder for this preprint (which was not certified by peer review) is the author/funder, who has granted bioRxiv a license to display the preprint in perpetuity. It is made available under aCC-BY 4.0 International license.

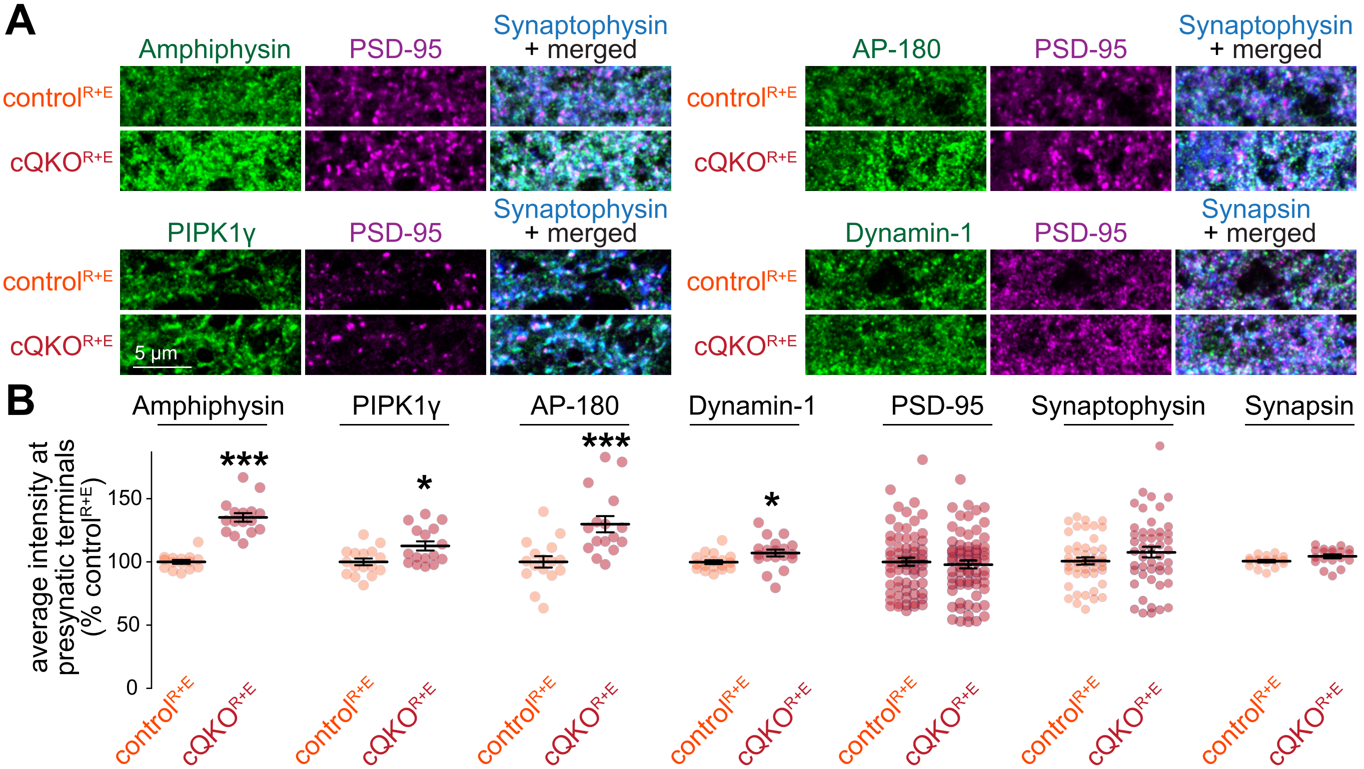

bioRxiv preprint doi: https://doi.org/10.1101/2025.04.23.650151; this version posted February 26, 2026. The copyright holder for this preprint (which was not certified by peer review) is the author/funder, who has granted bioRxiv a license to display the preprint in perpetuity. It is made available under aCC-BY 4.0 International license.

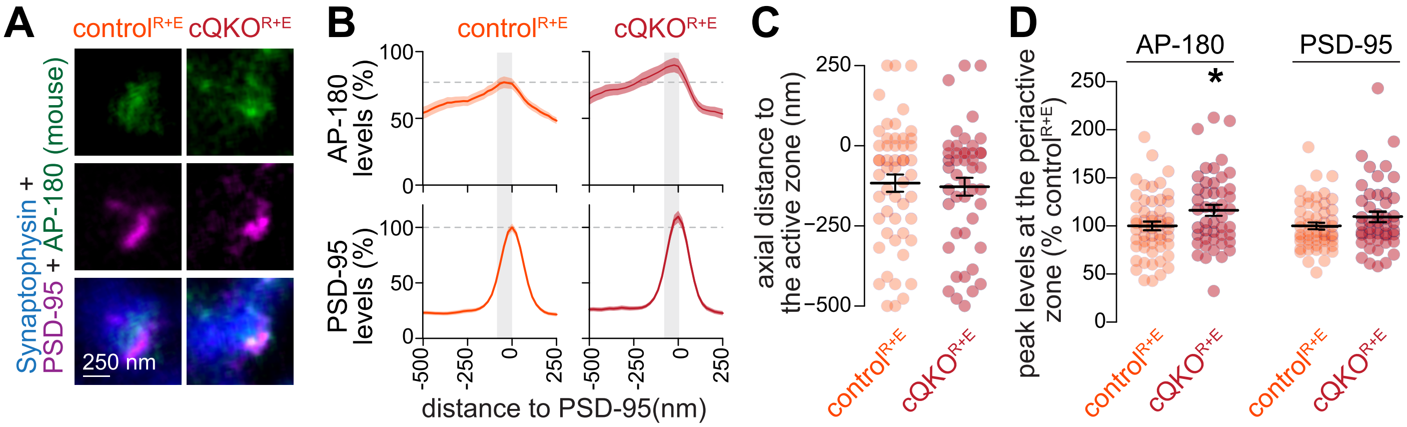

bioRxiv preprint doi: https://doi.org/10.1101/2025.04.23.650151; this version posted February 26, 2026. The copyright holder for this preprint (which was not certified by peer review) is the author/funder, who has granted bioRxiv a license to display the preprint in perpetuity. It is made available under aCC-BY 4.0 International license.

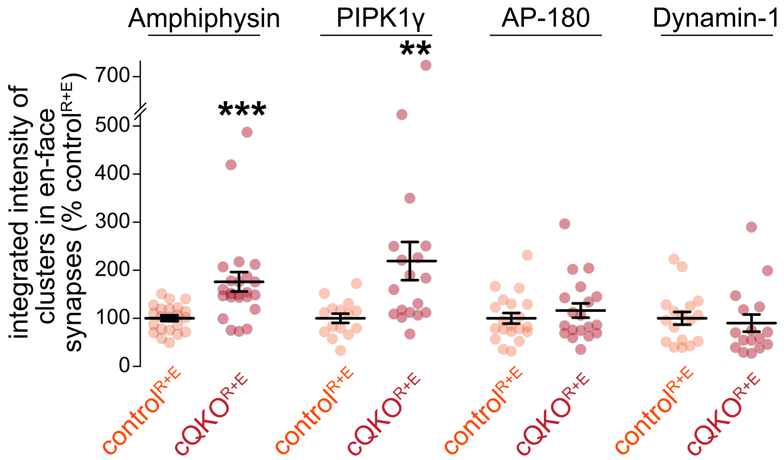

bioRxiv preprint doi: https://doi.org/10.1101/2025.04.23.650151; this version posted February 26, 2026. The copyright holder for this preprint (which was not certified by peer review) is the author/funder, who has granted bioRxiv a license to display the preprint in perpetuity. It is made available under aCC-BY 4.0 International license.

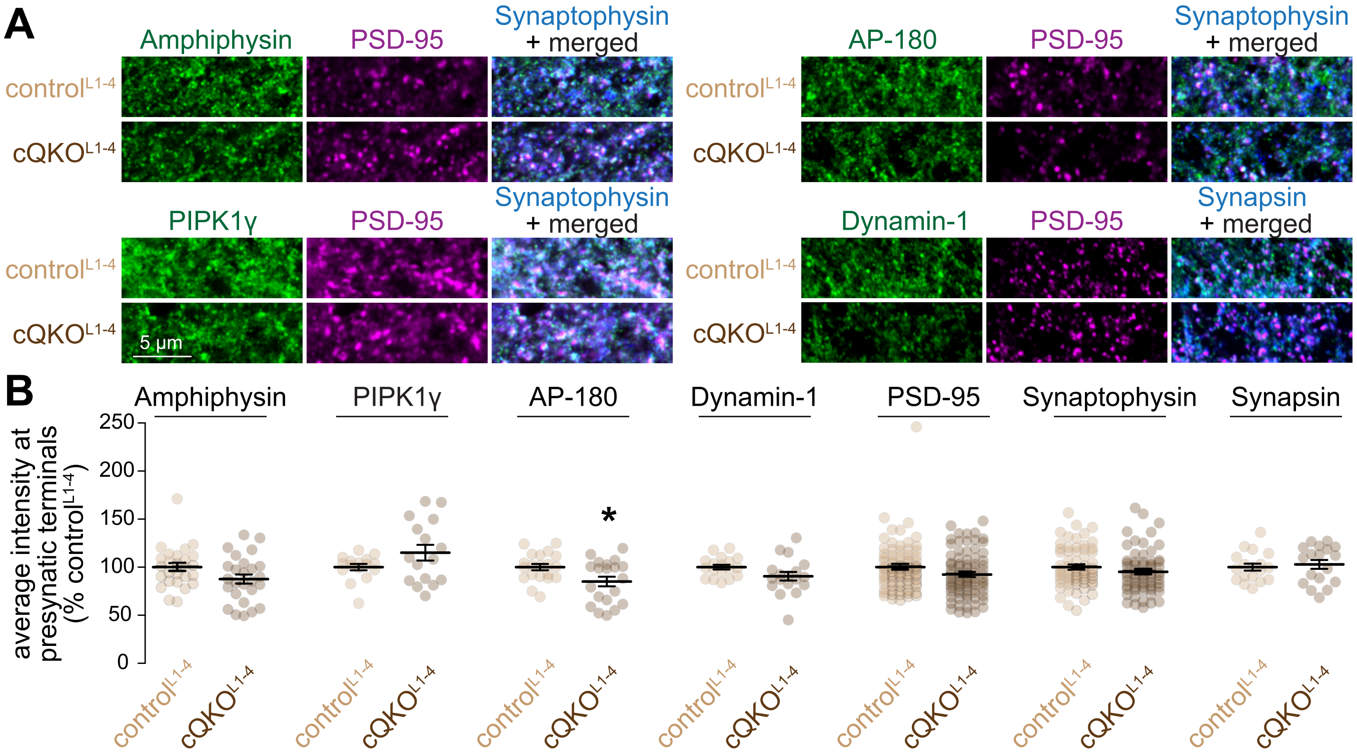

bioRxiv preprint doi: https://doi.org/10.1101/2025.04.23.650151; this version posted February 26, 2026. The copyright holder for this preprint (which was not certified by peer review) is the author/funder, who has granted bioRxiv a license to display the preprint in perpetuity. It is made available under aCC-BY 4.0 International license.

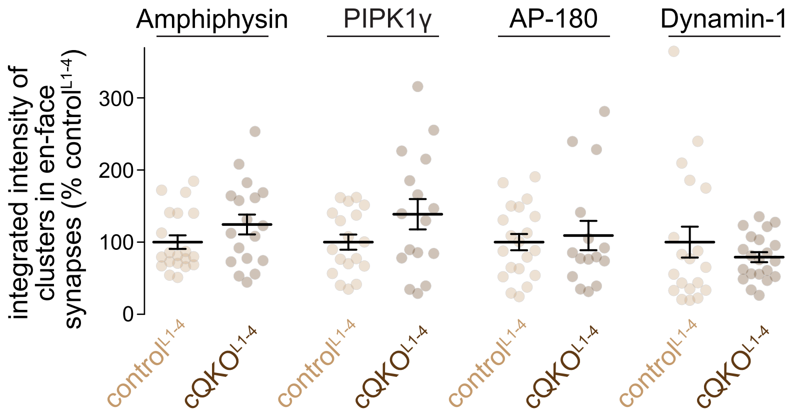

Supplement: Supplement 1 [file NIHPP2025.04.23.650151v2-supplement-1.pdf]
